# Techno-Economic Analysis and Life Cycle Assessment of High-Velocity Oxy-Fuel Technology Compared to Chromium Electrodeposition

**DOI:** 10.3390/ma16103678

**Published:** 2023-05-11

**Authors:** Antoine Merlo, Florin Duminica, Alain Daniel, Grégoire Léonard

**Affiliations:** 1 Department of Chemical Engineering, University of Liège, Quartier Agora B6a Sart-Tilman, 4000 Liège, Belgium; 2 Centre de Recherches Métallurgiques, CRMGroup, Avenue du Bois Saint-Jean, 21, 4000 Liège, Belgium

**Keywords:** HVOF, chromium electrodeposition, life cycle assessment, technology comparison, coatings

## Abstract

Due to the toxicity associated with chromium electrodeposition, alternatives to that process are highly sought after. One of those potential alternatives is High Velocity Oxy-Fuel (HVOF). In this work, a HVOF installation is compared with chromium electrodeposition from environmental and economic points of view by using Life Cycle Assessment (LCA) and Techno-Economic Analysis (TEA) for the evaluation. Costs and environmental impacts per piece coated are then evaluated. On an economic side, the lower labor requirements of HVOF allow one to noticeably reduce the costs (20.9% reduction) per functional unit (F.U.). Furthermore, on an environmental side, HVOF has a lower impact for the toxicity compared to electrodeposition, even if the results are a bit more mixed in other impact categories.

## 1. Introduction

Chromium coating is a vital process for many industries, such as the aerospace or the automotive sectors. This is due to the interesting properties that those types of coatings bring: reducing friction and increasing hardness, as well as wear and corrosion resistance [1]. As a consequence, this treatment allows one to improve the life time of the pieces coated as well, making the pieces more reliable when in use [2]. However, the most common technology used to make those types of coatings is chromium electrodeposition. That technology is quite popular due to its advantages of having low investment and consumable costs, ease of implementation, and relatively low energy consumption compared to other coating technologies. However, one of the drawbacks of the technology is its use of hexavalent chromium compounds, which have a strong carcinogenic effect and have been shown to increase the risks of lung cancer in the workers of electrodeposition plants [3]. This is due to the emission of droplets during the electrodeposition process caused by bubble formation with the electrolysis of the plating solution. Furthermore, the supply chain of the chromium compounds used in the electrodeposition process is also responsible for a lot of hexavalent chromium emissions along the chain [4]. Hexavalent chromium has been infamously present in a number of environmental and health cases, the most famous of which has been adapted to the big screen in the movie Erin Brockovich and involves the contamination of groundwater near Hinkley, California in 1993 by the Pacific Gas and Electric company, which has led to inhabitants developing cancer [5]. Additionally, installations of chromium electroplating have been demonstrated to lead to soil contamination in the surrounding area [6]. There is then a pressure on industry to either invest large amounts of money in preventive measures to abate the hexavalent chromium emissions, or to use alternative technologies to chromium electrodeposition [7]. Indeed, while currently chromium trioxide is used in the EU at an annual rate of 7000 tons, the use of this compound is technically forbidden unless an authorization is granted by the European Commission. Such authorizations have been given for electroplating industries, but are set to expire in September 2024 [8]. While these authorizations can be renewed, the European Parliament took legal action against the European Commission over granting those authorizations [9]. This makes the future of chromium electroplating uncertain in the EU and might make alternatives to the process a necessity rather than a preference. As such, it is required to assess those alternatives on an economical and environmental basis to evaluate their viability.

In order to assess technologies regarding their environmental and economic performances, a high number of assessment technologies exist. Among those, LCA, for environmental impact, and TEA, for economic performances, are often regarded as part of the best assessment methods to be used in conjunction for a combined assessment. LCA consists in evaluating the environmental impact of a product or process by evaluating its emissions at all production steps using a process inventory [10]. An emission factor is then applied to those emissions to obtain an impact score. As for TEA, the goal is to give technical indicators and to evaluate the cost of the process or product by calculating the capital and operational expenditures of that process [11]. By using a common inventory for both these assessment methods, a synergetic approach is possible in order to make an integrated environmental and economic assessment. For example, in recent years, standardization of their combined use has started to emerge with the publication of guidelines for their combined use [12,13].

In a previous article [14], we had used LCA and TEA to assess the environmental and economic pertinence of using magnetron sputtering as an alternative to electrodeposition for chromium coatings. In some respects, magnetron sputtering is an advantageous technology, thanks to the absence of emissions during the deposition process, its lower dependence on labor costs, and the production chain of metallic chromium, which lowers hexavalent chromium emissions. However, other characteristics hold it back as a technology, such as its relatively low deposition rate and its high energy consumption [14].

In the present article, another alternative to electrodeposition will be explored on an economic and environmental basis: the High Velocity Oxy-Fuel (HVOF) process. That process could have the advantages of magnetron sputtering, i.e., close to no emissions during the deposition process and lower labor dependence compared to electrodeposition, but with added benefits: very high deposition rate and low electricity consumption. Although HVOF offers these advantages, the powders used in the process could have a high environmental impact, as well as the combustion of fuels during the process. Furthermore, those powders have a high cost, as well as the equipment needed for the process, which also requires high investment costs. As such, it is required to use assessment methods in order to determine if the advantages brought by the HVOF process are enough to compensate its potential downsides. This would allow us to find out if a relatively newer technology, such as HVOF, can compete with a more settled technology, such as chromium electrodeposition. A more detailed look into HVOF, as well as a review of the literature of the assessments, are made in the next section.

## 2. State-of-the-Art

### 2.1. Thermal Spraying and HVOF

Thermal spraying is a family of deposition techniques that relies on the projection of heated material towards a substrate in order to grow a coating. Thermal spraying was first developed in 1910 [15], starting with flame spraying in its original form, which involves the combustion of fuel gases to generate heat for melting a feed of particles, as well as expanding gases that will give speed to the melted particles. Those temperatures are usually around 2700 °C, and particle speeds reach up to 100 m/s [16]. Since then, the newer variants of the technology still use the same basic principles, but now they require more advanced hardware. Thermal spraying technologies, which derive from flame spraying are: Detonation Gun (D-Gun) spraying, High-Velocity Oxy-Fuel spraying (HVOF), and High-Velocity Air–Fuel spraying (HVAF).

With detonation gun spraying, fuel gases and oxygen are confined in a tube, and the detonation is initiated using a spark. A high-pressure shockwave is then generated, and the droplets can be sent at higher temperatures (4000 °C) and much higher speeds (up to 1200 m/s) than with typical flame spraying. The detonation cycle can happen between 1 and 10 times per second, and thanks to the increased temperatures and speeds, better adherence and lower porosities than flame spraying coatings can be achieved [17,18].

**Figure 1:**
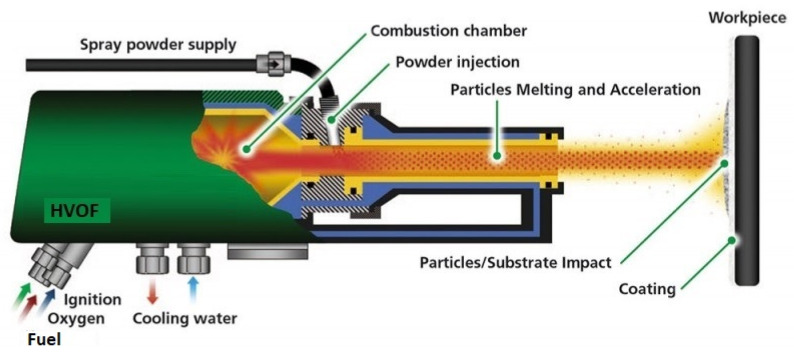
Representation of the HVOF process, credit to Flame Spray Technologies (FST).

With HVOF, the process relies on the combustion of a pressurized mixture of oxygen and fuel, which both come in a continuous gas flow. With a high flowrate and pressure, high temperature and supersonic speeds can be achieved (3000 °C and 1000 m/s respectively) [19]. Similarly to D-Gun spraying, good adherence and low porosity can be achieved compared to conventional flame spraying. HVOF comes in addition with the ability to attain higher deposition rates thanks to the continuous nature of the process. HVOF is usually performed with powders, but solutions and suspensions can be used instead of powders [20]. A representation of a HVOF gun can be seen in Figure 1.

HVAF is extremely similar to HVOF, except that oxygen is replaced with air. This has the advantage of being cheaper than HVOF, but due to the lower oxygen content, lower temperatures can be attained and so the range of materials HVAF can treat is smaller [21].

Other thermal spraying methods exist that do not involve combustion in the process. They can broadly be put into two categories: plasma spraying and kinetic spraying. Plasma spraying can involve different technologies, which each use plasma as an energy source. First, Atmospheric Plasma Spraying (APS) takes place at atmospheric pressure and uses a discharge to generate the plasma. The use of a plasma jet allows to reach much higher temperatures for the melt that is being projected on the substrate, being in the range of 10,000 to 30,000 °C [22], increasing the range of materials that can be melted. Second, solution or suspension precursor plasma spraying (SPS/SPPS), where the same principle is used, except the feed is in liquid state and finer powders can be used. Third, low-pressure or vacuum plasma spraying where the process takes place in a vacuum chamber and the “in-flight” interactions of the materials are limited. Additionally, finally, wire arc spraying, where a wire is fed to the process and its material is melted through the generation of an electrical arc, is observed.

Kinetic spraying, also called cold spraying, does not involve extensive heating of the feed, but instead relies on the transfer of large amounts of kinetic energy to the material in order obtain the conditions necessary to grow the coating. The gas used is itself hot. However, the particles remain in a solid state, rather than being melted. For these types of coatings, the feedstock has to be limited to certain materials that can meet the conditions necessary to be deposited in that state. However, the low temperatures allow lower residual stresses, as well as lower amounts of oxidation of the material during the deposition [23]. Typical temperatures for the gas are 400 °C to 800 °C, while, depending on the material and other factors, velocities can range from a few hundred m/s to 1400 m/s for their upper limit [24].

In this paper, the main focus will be put on HVOF, as it is one of the technologies most regarded as being a potential replacement to hard chromium deposition. The reasons for this will be developed further in the next section, and other potential replacements for the hexavalent chromium will also be discussed in a further section. On a technical side, HVOF has the capacity to deposit hard coatings, such as tungsten carbide in a cobalt matrix (from hereon called WC/Co), at high deposition speeds. Those coatings have been chosen due to their high performance in terms of hardness, toughness, and wear resistance [25], which is on par with, if not higher than, chromium coatings. Indeed, superior wear resistance has often been demonstrated in the literature of WC/Co compared to hard chromium [26,27,28]. Furthermore, as HVOF is not capable of depositing pure chromium due to its oxidation temperature [29], other types of coatings have to be studied. However, there might be some aspects that hold back HVOF as a technology. For instance, the investment costs are higher compared to electrodeposition, and the combustion of fuel and subsequent CO_2_ emissions add to the operational costs and environmental impact of the HVOF process. Additionally, HVOF cannot be used for some pieces, such as tubes, due to the requirement of a line of sight from the gun to the substrate. Finally, in the case of HVOF deposition of WC/Co, other additional challenges are faced as well: for example, the high amounts of energy are required to make tungsten carbides or to use cobalt, a rare and toxic metal. All of these factors can make the deposition of WC/Co with HVOF a less attractive process. An overview of the assessments already achieved in the literature will be presented. The goal of that overview is to obtain some insight about the HVOF process in order to identify the different upsides and downsides, both economic and environmental, of WC/Co deposited by HVOF compared to electrodeposition.

### 2.2. Previous Economic and Environmental Assessment of the HVOF Technology

**Figure 2:**
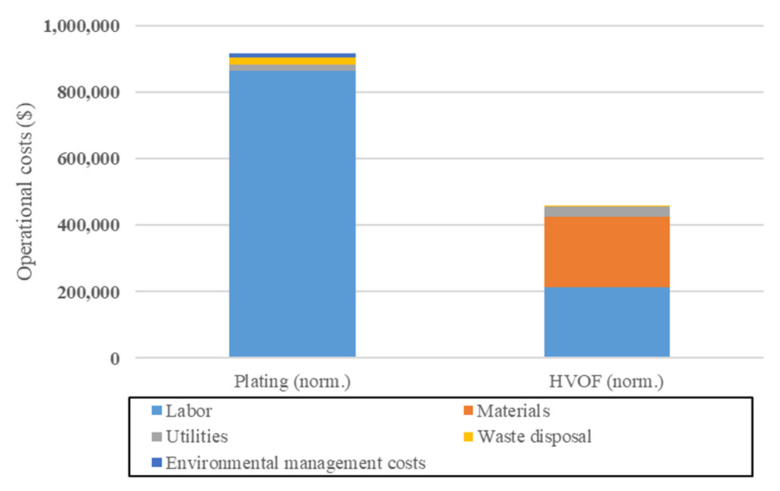
Comparisons of the yearly operational costs of HVOF and chromium electrodeposition. Adapted from [30].

As said previously, HVOF is widely regarded as a good option to replace chromium electrodeposition and is then relatively often evaluated based on its economic and environmental performances, even though rarely on both aspects simultaneously. For example, in the ESTCP Cost and Performance Report [30], Sartwell et al. aimed to assess, among other aspects, the operational costs associated with a partial switch from chromium electrodeposition to HVOF. The results obtained are quite encouraging for HVOF, showing a reduction of about 50% in the yearly operational costs compared to electrodeposition, as shown in Figure 2.

The main cost reduction comes from the reduction in labor cost, which shows about a four-fold reduction. Furthermore, although they are not very important, HVOF eliminates most costs associated to waste disposal (treatment of spent solution) and environmental management (abatement of emissions) that are present in Chromium electrodeposition processes. However, the materials costs increased by a wide margin, being about as costly as labor. This is due to the WC/Co powder used for HVOF being much more expensive compared to the reactants used in chromium electrodeposition. It should be noted that the yearly production volume for both technologies is uneven in the original publication, i.e., 65% of the yearly production was considered to be handled by HVOF, while 35% was considered to be handled by chromium electrodeposition. In Figure 2, annual costs have been linearly scaled to represent an annual production volume of 5500 pieces for each technology.

In another publication, from Krishnan et al., the environmental performances of HVOF and electrodeposition for landing gears have been compared [31]. LCA has been used for the analysis, and the results for the endpoints have been compared. Impacts for HVOF in the ‘Human Health’ and ‘Ecosystem Quality’ categories are clearly lower than for the electrodeposition process (between a factor 2 and 10 for ‘Human Health’ depending on the case, and several degrees of magnitude for ‘Ecosystem Quality’). This is due mostly to the toxicity of the compounds used in the electrodeposition process. On the other hand, results are more mixed for the ‘Resources’ category, being relatively equal for both processes, but for different reasons: the impacts for electrodeposition are mainly driven by electricity consumption, while the impacts of HVOF are mainly driven by powder production.

In another publication from Parker et al. [32], better mechanical performances were found for HVOF compared to electrodeposition, as well as better corrosion resistance in the atmospheric exposition test. However, corrosion resistance was equivalent for both technologies in the ASTM B117 test. Furthermore, a reduction in operational costs of about 20% was found with the usage of HVOF. This is a figure quite different from the 50% reduction obtained in [30]. However, a 50% reduction can also be obtained in [32], provided the stripping phase (removal of the old coating from the piece before the application of a new coating) for HVOF is not included, as is the case in [32].

Finally, in other publications from Sartwell et al. [33,34], HVOF coatings have been found to be better in terms of fatigue and leakage protection. Furthermore, HVOF has shown better corrosion resistance compared to electrodeposition. Additionally, finally, a much lower requirement of labor time was found compared to electrodeposition, explaining the labor cost reductions obtained in the previous studies.

As hard chromium plating has been developed for close to 100 years at the time of writing, with the first chromium coatings dating from the 1920′s [35], it is undeniably more mature than HVOF. Indeed, HVOF has only seen first experiments from the 1980s onwards [36]. This leads to HVOF competing with a more settled technology, such as hard chromium. That being said, HVOF has the potential to replace chromium electrodeposition for given applications. This is thanks to its novel ability to deposit materials, such as ceramics, at very high deposition speeds. To evaluate that potential, its performance needs to be evaluated.

The goal in the present work is, then, to evaluate, in parallel, the environmental and economic performances of HVOF compared to electrodeposition in order to determine its suitability as an alternative technology. HVOF has been selected due to its ability to give coatings with similar or better performance than hard chrome, its capacity to handle repair works and thicker coatings (>100 µm), its encouraging results in the literature, and the availability of an existing installation present in the CRMGroup to obtain primary data. The research gap, which the present work aims to fill, is the relatively low amount of combined environmental and economic studies on HVOF. To that end, the methods used for both types of assessment will be described in the next section. Then, environmental results will be given, followed by economic results. Finally, several scenarios for both technologies will be considered. More generally, this paper proposes an economic and environmental comparison between two competing deposition technologies. This methodology could then be easily applied to further case studies with the objective of providing some rational guideline for technology selection.

### 2.3. Other Potential Technologies for the Replacement of Hexavalent Chromium

Finally, it should be noted that HVOF is not the only technology well regarded for the replacement of hexavalent chromium electrodeposition. Many technologies have also been investigated during the years [37,38], with some of them being, in the same manner as HVOF, not reliant on a liquid medium, such as: magnetron sputtering [39], arc deposition [40], laser cladding [41], ion beam [42], or chemical vapor deposition [43]. All of these technologies have the capacity to produce coatings with interesting properties that allow them to be able to replace hexavalent chromium plating in some fashion, with high hardnesses and high wear resistances that allow them to take the place of chromium to coat newly produced pieces. In a lot of cases, however, there are some aspects of the technologies that hold them back as full replacement of hexavalent chromium. For example, with magnetron sputtering and other similar physical vapor technologies, large coating thicknesses cannot be realistically reached, and those technologies cannot, therefore, replace chromium plating for the purpose of coatings designed for the repair of damaged equipment. Other types of drawbacks can include, for example, coatings providing similar or better wear resistance, but not providing enough corrosion resistance, or requiring high temperatures for the growth of the coating, which can limit the choice of the substrate.

Another technology among the most promising to replace hexavalent chromium electrodeposition is trivalent chromium electrodeposition. Indeed, by replacing the hexavalent chromium salts with a non-toxic variant, the health problems can be totally avoided, all while keeping a relatively similar process. Using such types of baths, the use of lead anodes was not required, and large thicknesses of hundreds of micrometres have been reached, with similar or higher deposition rate as hexavalent solutions, as well as with similar or better hardness [44,45,46]. While such advantages would make trivalent chromium a strict upgrade to hexavalent chromium, unfortunately the process has some drawbacks, which render its adoption and implementation in the industry difficult. Those drawbacks include: the high voltage potential required for the reduction of Cr^+3^ to the metallic state, the stability of trivalent chromium compounds in aqueous solutions, and the quick precipitation of chromium hydroxides from the solution [47]. These drawbacks imply the requirement of a strict control of the composition and acidity of the bath where complexing agents have to be used in order to lower the potential of Cr^+3^ reduction and prevent the formation of stable compounds and of hydroxides. Furthermore, the coating obtained with trivalent plating is an alloy of chromium (for example, Cr-C), rather than pure metallic chromium, which can alter its properties, and the bath may then require additives, for example, to change the coloration of the coating. Therefore, the practicality of making thick coatings at a large scale using trivalent chromium can be compromised, as well as its ability to make repairs of damaged pieces. Nevertheless, trivalent chromium plating’s potential as a replacement to hexavalent chromium should not be underestimated, and it deserves its own sustainability assessment study. For now, the present work will focus on HVOF due to its proven ability to replace hexavalent chromium plating in a lot of applications.

## 3. Materials and Methods

LCA will be used in order to compare the environmental impacts of HVOF and chromium electrodeposition, while TEA will be used to compare the costs of the two technologies. For the construction of the inventory of chromium electrodeposition, the inventory from a previous article [14] is taken and can be seen in the Supplementary Materials S1. As for the construction of the inventory of the HVOF process, data from an existing installation available at CRMGroup are used.

### 3.1. Functional Unit

The functional unit, i.e., the unit of production for which the cost and environmental impacts will be assessed, is a coating of 1 m^2^ on a cylinder whose thickness will be 150 μm. The composition of the coating will be different for both technologies, using pure chromium for electrodeposition, as well as WC/Co (87% WC, 13% Co) for HVOF. An amount of 150 µm is in the range of film thicknesses typically produced by HVOF and by chromium electrodeposition. The performance of the two coatings will be considered equal for the purpose of the assessment, as even if there can be mechanical differences in favor of HVOF, they can be hard to quantify for the definition of a functional unit.

### 3.2. Goal and Scope

The goal is to assess and compare the environmental and techno-economic impacts for the coating of an object with two different deposition techniques and materials: hard chromium with electrodeposition and WC/Co with HVOF. The main impact categories considered will be global warming and toxicities, as they are the main sources of environmental impacts associated with those two technologies. Impact on resources will also be considered, as it has been shown that HVOF has a notable impact in that category [31]. Cost analysis is also conducted to highlight the economical aspect of those two deposition technologies.

For the purpose of the technological comparison, this study will focus on the deposition step itself and not the use phase of the coating nor on the cylinder manufacture. This is also known as a “cradle-to-gate” approach, where the production of the different input flows for the production step are taken into account, but not the final steps of the life cycle (disposal, recycling). Several recycling scenarios will be considered and will later be added to this initial cradle-to-gate assessment.

### 3.3. HVOF Process Inventory

As a reminder, the HVOF process inventory is largely based on data coming from an existing unit available at CRMGroup. The deposition phase for HVOF takes place in a ventilated cabin and consists in the combustion of a pressurized oxygen and ethanol mixture, which will heat, melt, and project the WC/Co powder. Emissions related to the production of oxygen and ethanol are taken into account, as well as CO_2_ emissions related to their combustion. As for the projected powder, it should be noted that all the projected powder does not end up on in the newly formed coating. Indeed, part of it is either reflected off the surface, or will not end up on the surface due to the angle of the jet so that only 50 to 75% of the powder projected is part of the coating (from hereon, an assumption of 50% is taken in order to consider the upper limit of emissions). That powder is considered to be recovered and reusable. The cabin is ventilated heavily in order to remove the WC/Co particles from the air. The air with the WC/Co goes through a filter so that particles are recovered from the air flow. That filtering is assumed to have a 99.9% abatement efficiency, meaning that 0.1% of the unused powder will be emitted. The characteristics of the deposition for a given functional unit are given in Table 1 for both HVOF and electrodeposition.

One point of attention is the difference in deposition speeds in both processes. This will have an impact on productivity further in the economic assessment. The process consumption and emissions per functional unit are also given in Table 2. The inventory for ED can be found in [10] and Supplementary Materials S1. Ethanol is fed as a liquid to the deposition gun, while oxygen is available as a compressed gas.

A complete ethanol combustion is assumed $\left({\mathrm{CH}}_{3}{\mathrm{CH}}_{2}\mathrm{OH}+3O_{2}\to 2{\mathrm{CO}}_{2}+3H_{2}O\right)$, meaning that the resulting CO_2_ emissions from the combustion amount to 21.19 kg CO_2_ per functional unit. For the purpose of this analysis, masking, sanding, and machining of the piece are omitted, and only the deposition is considered. Those sub-steps are indeed common to both processes and are not considered to have an important environmental impact relative to the deposition step due to their relatively low material and energy consumptions. The powder projected that is neither deposited, nor ends up as dust emissions, is considered to be recovered for further re-use without any need for additional treatment.

The energy demand for the production per kg of WC/Co powder is calculated based on the works of Kruzhanov [48] and amounts to 0.96 kWh per kg. The powder composition has been retrieved from the industrial process, leading to a mass ratio WC/Co of 87%/13%. Finally, the deposition process is assumed to take place in Belgium, so that, for both processes, electrical energy for deposition comes from the Belgian mix. For other energy consumptions, such as the energy required for the production of the powder, a global average is considered.

## 4. Results

### 4.1. Environmental Assessment

With the inventory obtained above, a LCA is conducted with the ReCiPe 2016 method in hierarchist configuration, the software Simapro 9.3.0.3, and the Ecoinvent 3 database is used.

**Figure 3:**
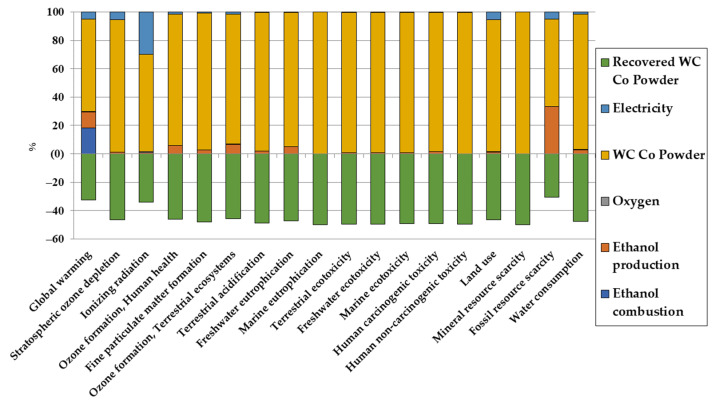
Repartition of the LCA results for the environmental impacts of HVOF per F.U. in midpoint categories.

**Figure 4:**
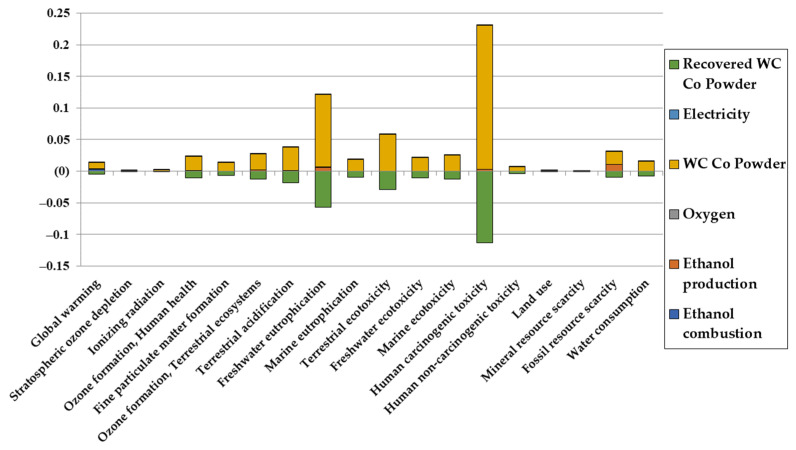
Normalized LCA results for the environmental impacts of HVOF per F.U. in midpoint categories.

Firstly, the impact assessment of the HVOF process is given in Figure 3 and Figure 4 for the repartition of the impacts and the normalized impacts, respectively. Normalized impacts are defined as the impact being divided by a normalization factor, which is, for ReCiPe 2016, the average global annual impact of an individual in 2010.

As one can see from the repartition of impacts, the main impact contributor in most categories is the production of the WC/Co powder. Before going into the details of the reasons for that behavior, a few more things are noted:

- There is a green negative value, which represents the avoided impact of recovering the powder losses. In subsequent graphs, that avoided impact is subtracted to the impact of the powder. It is, however, present in this case in order to showcase the impact of not recovering the lost powder;
- Ethanol production and combustion can be important contributors for greenhouse gas emissions, as the ethanol production path taken from the Ecoinvent database considers ethylene hydration. This assumption is taken in order to have an upper limit for the impact of the ethanol production for the HVOF process;
- Ethanol production is subsequently also a main contributor for fossil resource scarcity.

**Figure 5:**
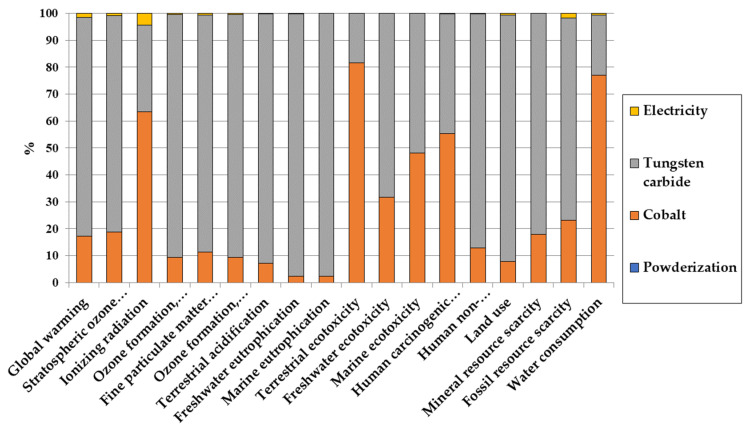
Distribution of the environmental impacts of WC/Co powder production.

To further explain the impact of the production of the tungsten carbide powder, a more detailed look into the impact of powder production is given in Figure 5 for the distribution of impacts.

The energy use and powderization itself play an almost negligible role in the environmental impact of the WC/Co powder production, while tungsten carbide and cobalt extraction and refining both have the most noticeable impact. This is due to the fact that both tungsten and cobalt are rare elements in the crust of the earth (1.25 ppm and 25 ppm, respectively [49]), and, as such, their extraction and refining lead to large energy demand and high emissions. Indeed, large volumes of ore need to be treated to obtain a given amount of these materials [50]. Due to the higher rarity of tungsten in the earth’s crust, its higher concentration in the WC/Co powder (87% of the powder is WC) and the energy required to react tungsten with carbon to synthetize WC, it is overall the main contributor to most impacts.

Two observations can be made on the impacts of both cobalt and WC production:

- Eutrophication is a relatively important impact category dominated by WC production. This comes from the fact that tungsten is refined from ammonium paratungstate ((NH_4_)10(H_2_W_12_O_42_))·4H_2_O → 12 WO_3_ + 10 NH_3_ + 10 H_2_O), and the resulting ammonia has a strong eutrophication power [51];
- There are some categories in which cobalt is disproportionally impactful in respect to its concentration in the WC/Co powder. Most of them are toxicities, which is mainly due to the toxicity of cobalt.

**Figure 6:**
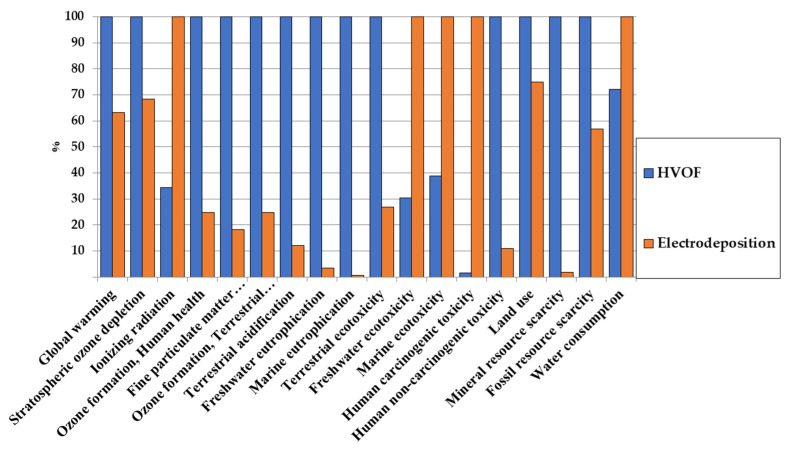
Comparison of the environmental impacts of HVOF and chromium electrodeposition.

**Figure 7:**
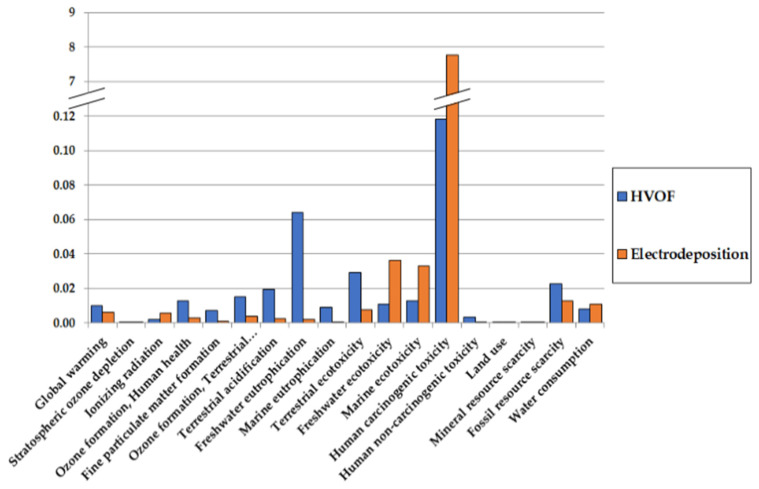
Comparison of the normalized environmental impacts of HVOF and chromium electrodeposition.

With the distribution of impacts given in the previous section, comparison with chromium deposition can be made. The comparison for one F.U. is given in Figure 6 and Figure 7 for comparison and normalized impacts, respectively.

In most impact categories, HVOF tends to have a worse impact than electrodeposition due to tungsten and cobalt production. However, as it can be clearly seen in Figure 7 and despite the toxicity of cobalt, the impact of HVOF for human carcinogenic toxicity is negligible compared to the impact of electrodeposition. It should be mentioned that results for metals toxicities should be taken carefully [52], but it is, however, encouraging for HVOF, as it would allow us to alleviate greatly the health impacts compared to electrodeposition.

Furthermore, as most of the impact comes from the extraction of powder materials, powder from recycled materials could be an effective way to reduce even further environmental impacts for HVOF technology. In the Supplementary Materials S2, a prospective analysis of recycling for HVOF is given. In the following section, an economic analysis is performed in order to assess the feasibility of HVOF.

### 4.2. Economic Assessment

The economic performance of the HVOF installation is assessed in this section. The following aspects are taken into account:

- Labor costs
- Operational costs
- Annuities on the initial equipment cost
- Maintenance

The annual production of the installation is assessed in order to work out the total cost per functional unit. A series of hypotheses consistent between the two processes are assumed, such as 300 working days per year, consisting in a single shift of 8 h per day. For HVOF, a spraying time of 34 min is necessary for the coating production, and a downtime of 20 min between the treatment of each piece to prepare the next spraying is assumed based on the typical usage at the CRMGroup facility. With those conditions, 2667 pieces can be treated annually.

As for electrodeposition, deposition lasts for 6 h, separated by 30 min of preparation between each deposition. As such, only 369 pieces can be deposited per year per bath. To arrive at the same productivity as the one of a HVOF installation, more than seven electrodeposition baths would be required. In order to have similar yearly productivities between HVOF and electrodeposition, seven electrodeposition baths are thus assumed, which is a number of baths consistent with existing small- to medium-scale plating operations.

According to CRMgroup, the total investment cost for the HVOF installation (robotic arm, HVOF gun, deposition cabin, and ventilation and control systems) is about 1 M€. As for the electrodeposition baths, a 37,500€ EUR cost per electrodeposition unit is assumed according to our previous work [14]. With seven baths, a total investment cost of 262,500€ EUR would be required if a linear increase in the cost with the number of baths is assumed. This figure will be kept as the investment costs for electrodeposition. However, there might be non-linear scale factors that would benefit electrodeposition, for example with common pieces of equipment that could be used for several baths (such as rinsing baths or scrubbers or transformers). For now, a linear increase in costs with the numbers of baths is assumed. For both HVOF and electrodeposition, a 10-year system lifetime is assumed, and maintenance costs have been estimated at 2% of the initial equipment costs per year. In the present study and for the considered functional unit, it has been assumed that working capital and plant infrastructure are not taken into account. Indeed, working capital does not affect the economic viability of a project, as it does not depreciate, while plant infrastructure is neglected because its impact is supposed to be similar for both processes.

According to CRMGroup, the labor required for a constant operation of the HVOF installation would require two operators. Concerning electrodeposition, one operator per bath could then be assumed. For both processes, it might be an overestimation of the amount of labor required. However, that makes the two processes more resilient in the face of unexpected issues in the workplace. Hourly wage is assumed at 40€ EUR/h [53].

Costs for the consumables are the following (based on costs applicable in 2021 at CRMGroup):

- WC/Co powder: 70€/kg
- Ethanol: 1.7€/L
- Oxygen: 17€/Nm^3^
- Electricity: 0.06€/kWh

**Figure 8:**
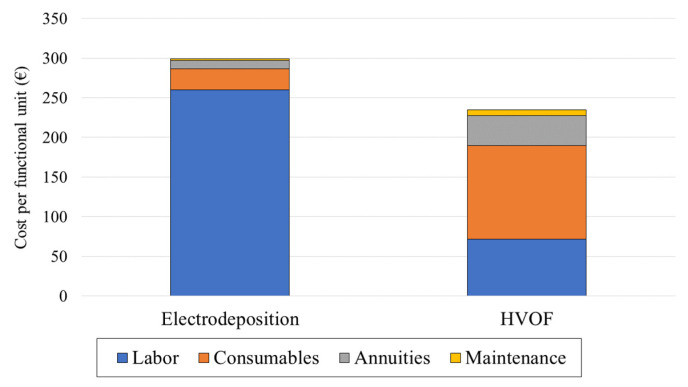
Distribution of the costs for electrodeposition and HVOF.

With all the above assumptions, the total yearly costs and cost per piece for the two technologies can be worked out. Annual costs for HVOF would then be 630,750€ EUR, leading to 236.5€ EUR per F.U. On the other hand, annual costs would be 772,130€ EUR for electrodeposition and 298.9€ EUR per F.U. The distribution of the costs is given in Figure 8 for HVOF and chromium, respectively.

### 4.3. Discussion

From this environmental and economic assessment, as a replacement for electrodeposition, HVOF seems very promising: indeed, on an economical level, the cost per piece is 20.9% lower with HVOF compared to electrodeposition (236.5 vs. 298.9€ EUR), thanks to the lower labor requirements. It comes at the cost of a higher upfront investment (1,000,000€ EUR compared to 262,500€ EUR for the equipment cost for HVOF and electrodeposition, respectively) for the HVOF installation, but that investment could be quickly recovered thanks to the lower operational costs. The results obtained are consistent with the results found in the literature, and, discussed in a previous section, similar savings for labor costs, as in [16], have been obtained. While the difference in costs is not as pronounced in the current work (20.9%) compared to [30] (50.1%, if normalized), it can be explained with differences in consumables costs, which can be attributed to either regional, temporal, or supplier differences, which influence the consumables costs. Furthermore, the costs considered in [30] are only operational, while the present study also includes equipment costs in the total cost calculations.

**Figure 9:**
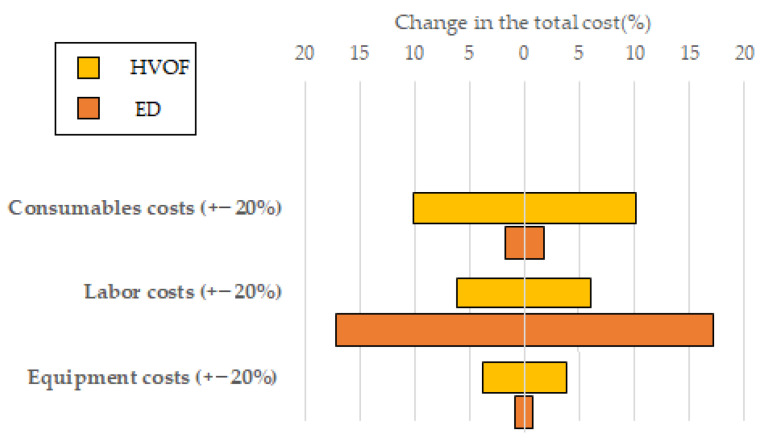
Influence of variations in cost categories on the total cost of the HVOF and electrodeposition processes.

In the Figure 9 below, a sensitivity analysis on the main cost categories (equipment, labor, and consumables) has been performed in order to demonstrate the influence of variations (between −20% and +20%) in the individual cost categories on the total cost.

Unsurprisingly, as the costs for the electrodeposition process are mainly driven by labor, variations in the cost of labor will heavily influence the total cost for electrodeposition compared to the HVOF process, as can be seen in Figure 9. This means that, while HVOF seems to be a more economically advantageous process, in countries with a lower labor cost, the results could be more in favor of electrodeposition. On the other hand, as the consumables cost are mainly responsible for the total cost of HVOF, variations in that category will influence the cost of the HVOF process much more than the electrodeposition process. This means that the process is less resilient to changes in the market price of the consumables, mainly the price of the WC/Co powder.

On an environmental level, it appears that, while there are tradeoffs in other impact categories and despite the toxicity of cobalt, HVOF will greatly improve the main problem linked to electrodeposition: its human carcinogenic toxicity. These results are similar to Krishnan, as well as with similar results for resources category. A point where results are different is the results on ecosystem quality, which are not as pronounced in favor of HVOF in the present study. This can be explained by the inclusion of waste disposal in Krishnan et al.’s study, which would increase electrodeposition’s impact. Furthermore, it should be noted that a lot of the environmental impacts come from the extraction of the materials, so that recycling could greatly reduce the impacts of both electrodeposition and HVOF. How and to which extent recycling could be performed is explored briefly in the Supplementary Materials S2.

Finally, on a technical level, HVOF can treat the same thicknesses range as electrodeposition and, for the same applications, it would be quite suited as a replacement. It can then also be used for repair works. Furthermore, many publications have found similar or better properties for HVOF coatings in terms of friction [54,55], hardness [26,32,54,55], fatigue strength [26], wear resistance [26,32,54,55], and corrosion resistance [26,30,32]. As such, it should meet requirements from manufacturers. However, as HVOF is a technology, which requires line of sight, some pieces, such as tubes or more complex pieces, might not be treatable by HVOF, and other alternatives would have to be used. Additionally, HVOF would necessarily have to coat thick coatings and could not handle thinner decorative coatings. A recapitulative table of the characteristics of HVOF WC/Co coatings and hard chromium coatings is presented in Table 3.

Interesting perspectives to this work would be to include the comparison of different alternatives to hexavalent chromium electrodeposition, such as other PVD techniques, trivalent chromium electrodeposition, or CVD, which would give an indication towards which technologies would be the most interesting in terms of economic and environmental performances.

## Figures and Tables

**Table 1:** HVOF and electrodeposition (ED) deposition parameters.

Characteristic	Quantity (HVOF)	Quantity (ED)	Unit
Deposition time	34	360	min
Coating density	8.5	7.02	g/cm^3^
Coating thickness	150	150	µm
Surface	1	1	M^2^
Deposited matter	1.28	1.05	kg

**Table 2:** HVOF consumption and emissions per functional unit.

Consumption per F.U.	Quantity	Unit
Ethanol	14.17	L
Oxygen	0.283	Nm^3^
WC/Co powder	2.55	kg
Electricity	22.67	kWh
Non-deposited powder	1.27	kg
Powder dust emissions	1.27	g

**Table 3:** HVOF and electrodeposition (ED) coatings parameters.

Characteristic	HVOF	Hard Chrome	Source
Friction coefficient (-)	0.37	0.70	[55]
Hardness (HV)	1240	800–1000	[55]
Fatigue strength (MPa)	900	840	[26]
Median wear rate (grams lost per abrasion cycle)	1.89	2.91	[26]
Corrosion resistance (corrosion after two days)	50%	100%	[26]

## Data Availability

Data supporting reported results can be found in the Supplementary Materials document: life cycle inventory of electrodeposition process.

## Supplementary Materials

The following supporting information can be downloaded at: https://www.mdpi.com/article/10.3390/ma16103678/s1. S1—Electrodeposition inventory contains the inventory of the chromium electrodeposition process. S2—Assessment of different scenarios contains the environmental results of different scenarios. Refs. [56,57,58,59] are cited in Supplementary Materials.

## References

[1] Gawne D.T., Ma U. (1989). Friction and wear of chromium and nickel coatings. Wear.

[2] Kohlscheen J., Knoche H.J., Hipke M., Lümkemann A. (2010). Coating development for gear cutting tools. Key Engineering Materials.

[3] Gibb H.J., Lees P.S.J., Wang J., Grace O’Leary K. (2015). Extended followup of a cohort of chromium production workers. Am. J. Ind. Med..

[4] Harscoet E., Froelich D. (2008). Use of LCA to evaluate the environmental benefits of substituting chromic acid anodizing (CAA). J. Clean. Prod..

[5] McNeill L., McLean J., Edwards M., Parks J. (2012). State of the Science of Hexavalent Chromium in Drinking Water. Water Res. Found..

[6] Sun J., Luo Y., Ye J., Li C., Shi J. (2022). Chromium Distribution, Leachability and Speciation in a Chrome Plating Site. Processes.

[7] ECHA (2016). Annex XVII to REACH—Entry 47.

[8] ECHA (2021). Chromium Trioxide Widely Used in Plating and Surface Treatment.

[9] Infocuria (2021). Action Brought on 5 March 2021–European Parliament v European Commission (Case C-144/21). https://curia.europa.eu/juris/document/document.jsf?text=&docid=240681&pageIndex=0&doclang=en&mode=req&dir=&occ=first&part=1&cid=2048529.

[10] Hauschild M.Z., Rosenbaum R.K., Olsen S.I. (2018). Life Cycle Assessment.

[11] Lauer M. (2008). Methodology guideline on techno economic assessment (TEA). Workshop WP3B Economics, Methodology Guideline.

[12] Van Schoubroeck S., Thomassen G., Van Passel S., Malina R., Springael J., Lizin S., Venditti R.A., Yao Y., Van Dael M. (2021). An integrated techno-sustainability assessment (TSA) framework for emerging technologies. Green Chem..

[13] Langhorst T., McCord S., Zimmermann A., Müller L., Cremonese L., Strunge T., Wang Y., Zaragoza A.V., Wunderlich J., Marxen A. (2022). Techno-Economic Assessment & Life Cycle Assessment Guidelines for CO2 Utilization (Version 2.0).

[14] Merlo A., Léonard G. (2021). Magnetron Sputtering vs. Electrodeposition for Hard Chrome Coatings: A Comparison of Environmental and Economic Performances. Materials.

[15] Siegmann S., Albert C. (2013). 100 years of thermal spray: About the inventor Max Ulrich Schoop. Surf. Coat. Technol..

[16] Davis J.R. (2004). Handbook of Thermal Spray Technology.

[17] Ke P.L., Wu Y.N., Wang Q.M., Gong J., Sun C., Wen L.S. (2005). Study on thermal barrier coatings deposited by detonation gun spraying. Surf. Coat. Technol..

[18] Singh L., Chawla V., Grewal J.S. (2012). A review on detonation gun sprayed coatings. J. Miner. Mater. Charact. Eng..

[19] Hoksa M., Turunen E., Suhonen T., Varis T., Hannula S.P. (2011). Optimization and characterization of high velocity oxy-fuel sprayed coatings: Techniques, materials, and applications. Coatings.

[20] Mittal G., Paul S. (2022). Suspension and solution precursor plasma and HVOF spray: A review. J. Therm. Spray Technol..

[21] Guo R.Q., Zhang C., Chen Q., Yang Y., Li N., Liu L. (2011). Study of structure and corrosion resistance of Fe-based amorphous coatings prepared by HVAF and HVOF. Corros. Sci..

[22] Chang J., Zhou Y.L., Zhou Y. (2011). Chapter 2—Surface modification of bioactive glasses. Bioactive Glasses: Materials, Properties and Applications.

[23] Herman H., Sampath S., McCune R. (2000). Thermal spray: Current status and future trends. MRS Bull..

[24] Assadi H., Kreye H., Gärtner F., Klassen T.J.A.M. (2016). Cold spraying–A materials perspective. Acta Mater..

[25] He J., Schoenung J.P. (2002). A review on nanostructured WC-Co coatings. Surf. Coat. Technol..

[26] Nascimento M.P., Souza R.C., Miguel I.M., Pigatin W.L., Voorwald H.J.C. (2001). Effects of tungsten carbide thermal spray coating by HP/HVOF and hard chromium electroplating on AISI 4340 high strength steel. Surf. Coat. Technol..

[27] Krelling A.P., de Souza M.M., da Costa C.E., Milan J.C.G. (2018). HVOF-sprayed Coating Over AISI 4140 Steel for Hard Chromium Replacement. Mater. Res..

[28] Sahraoui T., Fenineche N.-E., Montavon G., Coddet C. (2004). Alternative to chromium: Characteristics and wear behavior of HVOF coatings for gas turbine shafts repair (heavy-duty). J. Mater. Process. Technol..

[29] Schmid B., Aas N., Grong O., Odegard R. (2001). High-temperature oxidation of nickel and chromium studied with an in-situ environmental scanning electron microscope. Scanning.

[30] Sartwell B.D., Legg K.O., Kestler R., Nardi A., Haataja G., Guillemette R., Luchenta R., Mason A., Kaltenhauser P., Betz R. (2009). Replacement of Chromium Electroplating on Helicopter Dynamic Components Using HVOF Thermal Spray Technology.

[31] Krishnan N., Vardelle N., Legoux J.-G. A life cycle comparison of hard chrome and thermal sprayed coatings: A case example of aircraft landing gears. Proceedings of the International Thermal Spray Conference.

[32] Parker D., Sartwell B., Legg K., Schell J., Sauer J., Natishan P., Dull D., Falkowski J., Bretz P., Devereaux J. (2004). Validation of HVOF WC/Co Thermal Spray Coatings as a Replacement for Hard Chrome Plating on Aircraft Landing Gear. DTIC Document.

[33] Sartwell B.D., Legg K.O., Bodger B. Hvof thermal spray coatings as an alternative to hard chrome plating on military and commercial aircraft. Proceedings of the AESF/EPA Conference for Environmental Excellence.

[34] Sartwell B.D., Legg K.O., Bretz P. Status of HCAT/JG-PP program on replacement of hard chrome plating with HVOF thermal spray coatings on landing gear. Proceedings of the AESF Aerospace Plating and Metal Finishing Forum.

[35] Dubpernell G. (1984). History of Chromium. Plat. Surf. Finish..

[36] Kuroda S., Kawakita J., Watanabe M., Katanoda H. (2008). Warm spraying—A novel coatingprocess based on high-velocity impact of solid particles. Sci. Technol. Adv. Mater..

[37] Navinšek B., Panjan P., Milošev I. (1999). PVD coatings as an environmentally clean alternative to electroplating and electroless processes. Surf. Coat. Technol..

[38] Legg K.O., Graham M., Chang P., Rastagar F., Gonzales A., Sartwell B. (1996). The replacement of electroplating. Surf. Coat. Technol..

[39] Laxane R.B., Bhide R.S., Patil A.S., Sane S.G. (2006). Characterisation of chromium nitride physical vapour deposition coating on diesel engine pistons. Surf. Eng..

[40] D’Avico L., Beltrami R., Lecis N., Trasatti S.P. (2018). Corrosion behavior and surface properties of PVD coatings for mold technology applications. Coatings.

[41] Lampa C., Smirnov I. (2019). High speed laser cladding of an iron based alloy developed for hard chrome replacement. J. Laser Appl..

[42] Treglio J.R., Perry A.J., Stinner R.J. (1995). Ion beams replace chrome plating. Adv. Mater. Process..

[43] Nouvellon C., Belchi R., Libralesso L., Douhéret O., Lazzaroni R., Snyders R., Thiry D. (2017). WC/C: H films synthesized by an hybrid reactive magnetron sputtering/Plasma Enhanced Chemical Vapor Deposition process: An alternative to Cr (VI) based hard chromium plating. Thin Solid Film..

[44] Hong G., Slow K.S., Zhiqlang G.A.K. (2001). Hard chromium plating from trivalent chromium solution. Plat. Surf. Finish..

[45] Protsenko V.S., Danilov F.I. (2014). Chromium electroplating from trivalent chromium baths as an environmentally friendly alternative to hazardous hexavalent chromium baths: Comparative study on advantages and disadvantages. Clean Technol. Environ. Policy.

[46] Li B.S., Lin A. (2008). Study of Hard Chromium Plating from Trivalent Chromium Electrolyte. Key Eng. Mater..

[47] Benaben P. (2011). An overview of hard chromium plating using trivalent chromium solutions. Plat. Surf. Finish..

[48] Kruzhanov V., Arnhold V. (2012). Energy consumption in powder metallurgical manufacturing. Powder Metall..

[49] Haynes W.M., Lide D.R., Bruno T.J. (2016). CRC Handbook of Chemistry and Physics.

[50] Norgate T., Haque N. (2010). Energy and greenhouse gas impacts of mining and mineral processing operations. J. Clean. Prod..

[51] Leoni B., Patelli M., Soler V., Nava V. (2018). Ammonium transformation in 14 lakes along a trophic gradient. Water.

[52] Pizzol M., Christensen P., Schmidt J., Thomsen M. (2011). Eco-toxicological impact of “metals” on the aquatic and terrestrial ecosystem: A comparison between eight different methodologies for Life Cycle Impact Assessment (LCIA). J. Clean. Prod..

[53] StatBel (2022). Labour Cost-Labour Cost Is Highest in the Energy Sector. https://statbel.fgov.be/en/themes/work-training/wages-and-labourcost/labour-cost.

[54] Ham G.S., Kreethi R., Kim H.J., Yoon S.H., Lee K.A. (2021). Effects of different HVOF thermal sprayed cermet coatings on tensile and fatigue properties of AISI 1045 steel. J. Mater. Res. Technol..

[55] Wang S., Ma C., Walsh F.C. (2020). Alternative tribological coatings to electrodeposited hard chromium: A critical review. Trans. IMF.

[56] Lackner K.S., Azarabadi H. (2021). Buying down the Cost of Direct Air Capture. Ind. Eng. Chem. Res..

[57] Furberg A., Arvidsson R., Molander S. (2019). Environmental life cycle assessment of cemented carbide (WC-Co) production. J. Clean. Prod..

[58] De Visscher J.-L. (2019). Informal Interview about Chromium Plating in the Company le Chromage Dur.

[59] Kojima T., Shimizu T., Sasai R., Itoh H. (2005). Recycling process of WC-Co cermets by hydrothermal treatment. J. Mater. Sci..

