# Supplementary material for: Techno-Economic Analysis and Life Cycle Assessment of High-Velocity Oxy-Fuel Technology Compared to Chromium Electrodeposition
Source: Materials (Basel). 2023 May 11;16(10):3678. doi: 10.3390/ma16103678 (PMC10223351; doi:10.3390/ma16103678)
Supplement: Supplementary file 1 [file materials-16-03678-s001.zip › materials-2342891-supplementary.pdf]

S – Supplementary materials S1: Life Cycle Inventory of chromium plating

1. LCI

Here, the life cycle inventory for chromium plating is given. It is based on the life cycle inventory given in [1]. A multiplicative factor of 7.5 has been applied to all inputs and emissions in order to arrive at the correct thickness (20μm in [1], 150μm in the present case). Inventory for plating per F.U. is shown in Table 1.

Table S1:Plating process inventory

FLOWS

**INPUTS**	Amount	Unit
ELECTRICITY	151.5	kWh
CHROMIUM OXIDE	2238.75	g
H2SO4	1.275	g
WATER	2490	L
**OUTPUTS**		
LUBRICATING OIL	210	g
AIRBORNE CRVI EMISSIONS	188.25	mg
WATERBORNE CRVI EMISSIONS	408.75	mg

Furthermore, in addition to the plating process, the production of the consumables required by the wastewater treatment is assessed. It is shown in Table 2.

Table S2: Consumables used for the wastewater treatment process

FLOWS

**INPUTS**	Amount	Unit
NAOH	195	g
SO2	135	g
H2SO4	405	g
MGO	217.5	g
WATER	142.5	L

S2 – Supplementary materials S2: Assessment of different scenarios

1. Potential technological improvements

Here, a series of potential changes to the processes are explored in order to assess the variations in the environmental and economic results. Some potential improvements are considered here:

- CO_2 capture
- Changes of electricity mix
- Recycling of the materials

1.1 CO_2 Capture

Due to the fact that the HVOF combustion happens in a controlled environment, which is ventilated, and because the gas is filtered from its powders, the result is a particle-less gas which has a higher concentration of CO_2 than the atmospheric concentration. These conditions might be suitable for the installation of a CO_2 capture unit beyond the filter. With such a unit, direct CO_2 emissions due to the combustion could be greatly abated. In practice, this might not be as interesting due to the large volumes of air processed by the ventilation unit compared to the amount of CO_2 emitted by the combustion of ethanol. Environmental results regarding CO_2 emissions were totally abated for HVOF, and they are given in Figure 1.

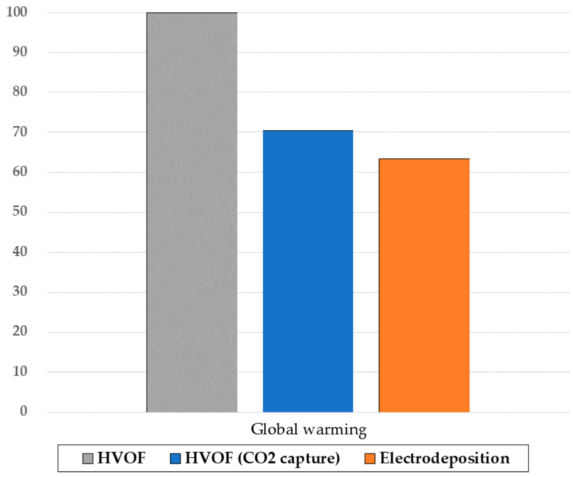

Figure S1: Comparison of the global warming potential of HVOF with CO_2 capture and electrodeposition

As one can see, HVOF is more competitive with electrodeposition in the GWP category with CO_2 capture compared to without. Indeed, the two processes now have almost equal global warming potential. Furthermore, even if direct emissions cannot be directly abated during the HVOF process, technologies, such as Direct Air Capture (DAC), could be used. With current commercial DAC plants being able to capture CO_2 at a cost of 600 $ USD/t[1], that capture cost could be marginal for the HVOF process (approximately 12 € EUR/F.U.). If DAC costs could go down even further with improvements in the technology, for instance to a value of 100 $/t[1], this would lead to around 2 € EUR/F.U.

1.2 Electricity mix

Another aspect that would change the environmental results would be the electricity mix used. Indeed, depending on how the electricity is produced, more or less CO_2 will be emitted for the same amount of electricity. In this case, as electrodeposition consumes a greater amount of electricity compared to HVOF, its environmental results will improve if the electricity source is decarbonized (both in absolute terms, as well as in a comparison with HVOF). To evaluate to which extent, two additional extreme cases are evaluated: the use of an electricity mix of 100% of coal combustion and an electricity mix of 100% of wind power. The results are compared to the base case of the Belgian electricity mix and are given in figure 2.

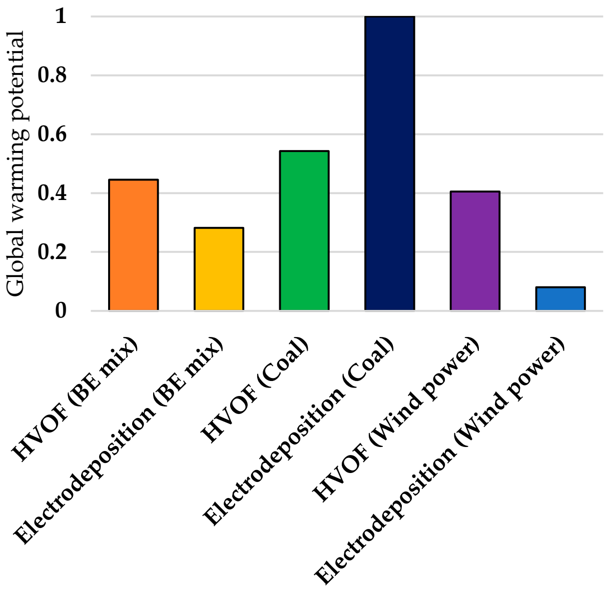

Figure S2: Comparison of HVOF and electrodeposition relative global warming potential with different electricity mixes

Overall, the worst impacts are for electrodeposition with electricity from coal. Its results become much worse in terms of global warming potential compared to HVOF. Additionally, while not negligible, the electricity mix does not drastically change the results of HVOF due to the relatively low usage of electricity of the technology, while the global warming potential of electrodeposition is strongly affected by the carbon intensity of the selected electricity mix.

1.3 Material recycling

As mentioned in section 4 of the related article, a lot of the impact is attributed to the powder used in the HVOF process. As such, recycling and recovery of the coatings could potentially reduce the environmental impacts by an important factor. One of the problems with the recovery of both chromium and HVOF coatings is that mechanical recovery (e.g., crushing, milling) is quite difficult due to the resistance and the hardness of the materials involved. Fortunately, other recovery routes are available.

For HVOF, the zinc recovery route from [2] is considered. In that route, molten zinc is applied to waste WC/Co coatings in their end of life, or to WC/Co from other sources. In this route, cobalt reacts with the coating by making a zinc–cobalt alloy in which the WC is still encapsulated. The zinc can then be extracted and reused, and a porous material is left over. That material containing the cobalt and tungsten carbide can then be crushed and milled into a powder that can be recompacted and sintered to be used for the HVOF process. In the worst case, recovery yield is 97%, and 1.3 kg of zinc and 4 kWh of electricity are required per kg of WC/Co. In the best case, recovery yield is 98%, and 1 kg of zinc and 1.5 kWh of electricity are required per kg of WC/Co. For both cases, losses to air of 0.5% of zinc are assumed, and lost WC/Co is solid waste [2].

For electrodeposition, recovery of chromium is quite simple: by using an invert current on a piece coated by chromium, the metallic chromium goes back to its ionic form in the bath, hence recovering the reactants. It should be noted that this technique comes with some drawbacks:

- Degradation of the plating solution and of the electrodes is accelerated.
- This leads to additional electricity requirements (not evaluated).
- As in the plating mode, droplets of the bath solution can be emitted through the formation of bubbles, potentially leading to hexavalent chromium emissions.

Unfortunately, little has been found in terms of data for reverse electroplating. According to the practitioners[3], that process takes less time and electricity than the standard electroplating. However, no values could be retrieved. As a consequence, two cases are assumed. In the best case, electricity consumption of the reverse plating is assumed to be one tenth of the standard electroplating electricity demand, and so are the hexavalent chromium emissions. In the worst case, electricity consumption of the reverse plating is one fourth of the standard electroplating, and so are the hexavalent chromium emissions. Recovery yield is assumed to be 98%.

For both cases, a formula for the impact of materials and recycling is used. A generic formula for recycling can be expressed as:

$$I_{TOT} = \frac{I_e + n(I_r + (1-\gamma)I_e)}{n+1}$$

Where:

- I_{TOT} is the total impact per functional unit.
- I_e is the impact of materials production from the extractive route per functional unit.
- n is the number of times the material is recycled.
- I_r is the impact of materials production from the recycling route.
- γ is the recycling yield.

Basically, this formula describes how the relative impact of the extraction of materials decreases with the number of recycling cycles (with $I_e/(n+1)$), the relative impact of the recycling process (with $nI_r/(n+1)$), and the compensation of losses in the recycling process by using new materials (with $(1 - \gamma)I_e$). For both processes, nine cycles are assumed, meaning a material is used 10 times (recycled nine times) before fully new material is used. This assumption taken in order to account for potential material degradation caused by the recycling process, with, for example, degradation of electrodes in the electrodeposition process and, hence, bath contamination or lowering of the mechanical properties of the recovered WC/Co material[4], which would ultimately need to be compensated by new materials or further processing.

With those considerations, environmental results with a Belgian mix are given in Figure 3.

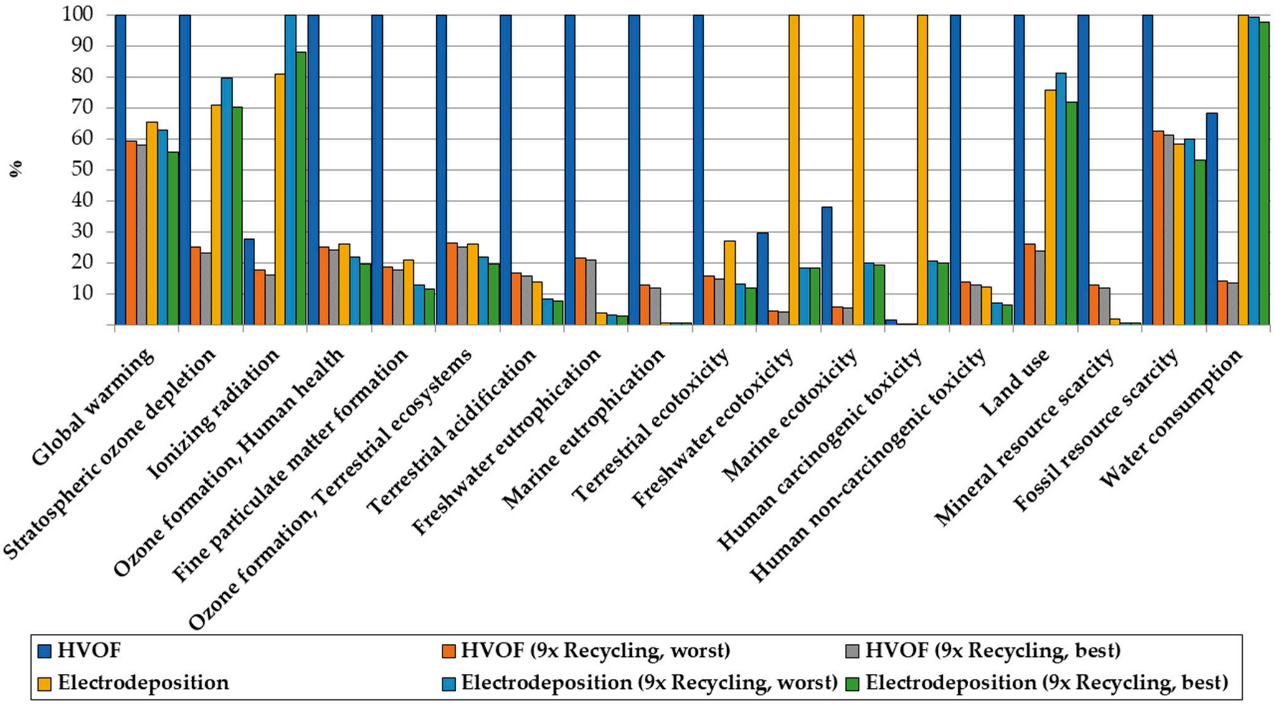

Figure S3: Comparison of HVOF and chromium electrodeposition in different recycling scenarios

Overall, HVOF benefits in a more pronounced way of the recycling processes due to the great relative impact of its materials. However, it should be noted that, for both technologies, the toxicities can be lowered drastically by recycling processes. In some categories, namely, stratospheric ozone depletion, ionizing radiation, land use, and fossil resource scarcity, the impact is higher for electrodeposition with the inclusion of recycling processes. This is due to the electricity used for the recovery of chromium oxide in the bath, those impact categories being mainly impacted by electricity use for electrodeposition. This would lead to increased impact in those categories, even with a lowered chromium use. The feasibility for both processes is yet to be completely proven. However, it is a good indication of the potential of recycling.

References

1. Lackner, K., S.; Azarabadi, H. Buying down the Cost of Direct Air Capture*. Ind. Eng. Chem. Res.* **2021**, 60(22), 8196-8208.

2. Furberg, A.; Arvidsson, R.; Molander, S. Environmental life cycle assessment of cemented carbide (WC-Co) production*. Journal of cleaner production*. **2019**, 209, 1126-1138

3. De Visscher, J.-L. Informal Interview about Chromium Plating in the Company le Chromage Dur, **2019**.

4. Kojima, T.; Shimizu, T.; Sasai, R.; Itoh, H. Recycling process of WC-Co cermets by hydrothermal treatment. *Journal of Materials Science*. **2005**, 40, 5167-5172
